# Subcutaneous implant with etonogestrel (Implanon®) for catamenial exacerbations in a patient with cystic fibrosis: a case report

**DOI:** 10.1186/1471-2466-14-165

**Published:** 2014-10-24

**Authors:** Adelaida Lamas, Luis Máiz, Marta Ruiz de Valbuena, José Manuel González-Casbas, Lucrecia Suárez

**Affiliations:** Department of Paediatric Pulmonology and Cystic Fibrosis Unit, Ramón y Cajal University Hospital, Carretera de Colmenar Km. 9,1, 28034 Madrid, Spain; Instituto Ramón y Cajal de Investigación Sanitaria (IRYCIS), Madrid, Spain; Department of Pulmonology and Cystic Fibrosis Unit, Ramón y Cajal University Hospital, Madrid, Spain; Department of Gynaecology, Ramón y Cajal University Hospital, Madrid, Spain; Department of Paediatric Gastroenterology and Cystic Fibrosis Unit, Ramón y Cajal University Hospital, Madrid, Spain

**Keywords:** Cystic fibrosis, Pulmonary exacerbations, Female hormones, Oral contraceptives, Hormonal contraception

## Abstract

**Background:**

Cystic Fibrosis (CF) is a genetic disease with equal prevalence across sexes. However, women present worse lung function with faster function decline, earlier onset of bacterial colonization, more frequent pulmonary exacerbations (PE), greater bronchial hyper-responsiveness, and higher mortality rates after puberty than men. The etiology of this gender disparity remains elusive but female hormones have been implicated in several studies.

**Case presentation:**

A 20-year-old female with CF with severe recurrent PE, always related to the menstrual cycle since menarche, combined with lung function decline requiring multiple courses of intravenous antibiotics. We report the cessation of PE and recovery of pulmonary function following the insertion of a subcutaneous implant with 68 mg of etonogestrel (Implanon®, Organon Española S.A. Laboratories, Madrid, Spain)**.**

**Conclusion:**

Our case report supports the key role of female hormones in the development of PE and in the decline of lung function in a woman with CF. When appropriate, hormonal manipulation through contraceptive methods should be considered as potential treatment.

## Background

Gender differences in the presentation of Cystic Fibrosis (CF) after puberty have been well described in the literature including a shorter median life expectancy by close to 3 years observed in women compared to men [1–4]. The etiology of this gender disparity remains unclear but several factors such nutritional status, CF-related diabetes, lung function (LF), airway microbiota, pulmonary exacerbations (PE), and sex hormones have been associated with it in previous studies [5, 6].

Estrogens (E) are involved in mucus secretion, lung development, expression of inflammation factors, and the risk of acquiring *Pseudomonas aeruginosa* (*P. aeruginosa*) including its conversion to the mucoid phenotype. In addition, E are also linked to increased activity of the sodium channel and Na^+^-K^−^ATPase, a decrease in the chloride secretion mediated by the calcium channel, reduced airway surface liquid (ASL) height, and a modification in the activity of the cystic fibrosis transmembrane conductance regulator (CFTR) [7–17]. Finally, the expression of progesterone (P) receptors in the cilia of airway epithelium is regulated by sex hormones. P decreases cilia beat frequency in human airway epithelial cells by approximately 40% and this reduced cilia beat frequency may, in turn, contribute to impaired mucociliary clearance (MCC) [18].

These findings suggest an association between the changes in female hormones related to patient’s menstrual cycle with the documented gender disparity in CF-related morbidity and mortality [10, 19]. Based on Irish register data, Chotirmall and colleagues reported that female CF patients on the oral contraceptive pill (OCP) required fewer antibiotic courses and had a lower PE rate than their counterparts not on OCP [8]. Sutton et al. [4] studied the effects of puberty on CF-related PE in women *versus* men by performing a cohort study using the United States Cystic Fibrosis Foundation Patient Registry. Post-puberty, the number of PE per year in males was 0.24 lower than in females. Below we present our favorable experience using a subcutaneous implant with etonogestrel (Implanon®) to reduce PE frequency and improve LF in a woman with CF.

## Case presentation

The patient was a 20-year-old female with CF, genotype R334W/Q890X and pancreatic sufficiency, diagnosed as a 3 month-old due to respiratory symptoms. Since diagnosis, the patient had had several PE caused by β-lactamase producer methicillin-susceptible *Staphylococcus aureus (S. aureus)*. At the age of 5, she experienced the first of many acute pancreatitis (AP) episodes secondary to several oral medications, including antibiotics. These episodes, associated with continuous PE, led to the insertion of a port-a-cath. Non mucoid *P. aeruginosa* isolations grew in five samples from April 2000 to September 2009 and three samples of *Stenotrophomonas maltophilia* were also collected, once in 2005 and twice in 2010. The patient was treated with intermittent aerosolized antibiotics (tobramycin and colistin) following the Spanish Protocol for eradication of *P. aeruginosa*, and was on azythromycin (500 mg, three times a week) from February 2007 to July 2009 [20]. Sputa acid-fast bacilli smears and cultures for mycobacterium isolates were consistently negative.

**Figure 1:**
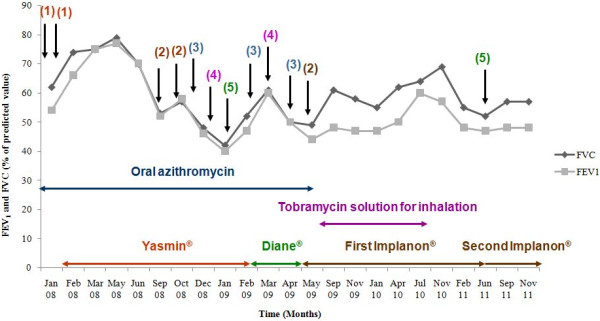
**Respiratory function and intravenous-antibiotic use before and after insertion of first and seconf subcutaneous implant with etonogestrel (Implanon®).** ⬇: intravenous antibic; FVC: forced vital capacity; FEV_1_: forced expired volumen in first second; (1): trimethoprim-sulfamethoxazole; (2) trimethoprim-sulfamethoxazole and linezolid; (3): linezolid; (4): trimethoprim-sulfamthoxazole and ceftazidimel; (5): Imipenem.

From the onset of menarche at the age of 14, PE had followed the same pattern. Four days before the menstrual period, she would start experiencing increased cough and purulent sputum production, develop daily fever (*>*38°C), exertional dyspnea, and anorexia. In February 2007, due to frequent PE and rapid LF decline, she was started on aerosolized ampicillin (Gobemicina®, Normon Laboratories, Madrid, Spain) every 12 hours. As previously reported [21], the response to this treatment was excellent for a year. On February 2008 she again suffered severe and frequent PE, even while on aerosolized ampicillin, and always related to menstrual cycle as described above. We then introduced an OCP with 3 mg of drospirenona and 30 mcg of etinilestradiol (Yasmin Diario®**,** Schering España S.A. Laboratories, Madrid, Spain) with initial good response and no PE for 7 months. On September 2008, the same pattern of PE described above re-started requiring five courses of intravenous (IV) antibiotics. From March through May of 2009, the patient was administered an OCP with 2.0 mg of ciproterone acetate and 0.035 mg of etinilestradiol (Diane 35®**,** Schering España S.A. Laboratories, Madrid, Spain). The lack of response called for four courses of IV antibiotics. In total, from February 2008 until May 2009 she required 20 weeks of IV antibiotics (ceftazidime, linezolid, imipenem, trimethoprim-sulfamethoxazole, or a combination of these; Figure 1). We observed a dramatic loss of LF as follows (Figure 1): forced expired volume in 1 second (FEV_1_) decreased from 2.33 L to 1.31 L (77% to 42% of FEV_1_ predicted) and forced vital capacity (FVC) decreased from 2.71 L to 1.49 L (79% to 42% of FVC predicted). In addition, she had five AP episodes. In May 2009, after discussing the case with the Department of Gynaecology, a subcutaneous implant with 68 mg of etonogestrel (Implanon®, Organon Española S.A. Laboratories, Madrid, Spain) was inserted in the inner side of her left arm. Estradiol levels had not been measured in this patient. Since then the patient had no PE episodes for two years, she did not require any oral or IV antibiotics, and her LF stabilized around 1.61 L for FEV_1_ (48% of FEV_1_ predicted) and 2.16 for FVC (57% of FVC predicted). In addition, the treatment resulted in a weight gain of 3 Kg (from 50 kg [10th percentile] to 53 kg [25th-50th percentile]). Further, no side effects were observed during this period, the pattern of *S. aureus* colonization did not change, and no selection of resistant microorganisms appeared except a unique isolation of non-mucoid *P. aeruginosa,* previously reported, four months after the insertion of the subcutaneous implant. From September 2009 through September 2010, we introduced aerosolized tobramycin (TOBI®, Novartis Laboratories) in on-off cycles following the Spanish Protocol for eradication of *P. aeruginosa*. In July 2011, the patient had a severe PE episode requiring treatment with IV antibiotic. At this point, the subcutaneous implant was replaced for another one. The patient has been PE episode-free since December 2012.

## Discussion

CF is a genetic disease with the same prevalence in both men and women. However, women experience worse LF, early onset bacterial colonisations, and lower survival expectancy than men [1–3, 22]. E levels are involved in mucus secretion, lung development, expression of inflammation factors, risk for *P. aeruginosa* colonization and its further conversion to mucoid phenotype, modification of the activity of the CFTR, and alterations of MCC and ion transport in the ASL [10, 15, 19, 23]. Additionally, P decreases cilia beat frequency in airway epithelial cells [18]. These effects have a negative impact on the MCC of the ASL which may explain, to an important extent, the lower survival in women with CF compared to their male counterparts.

All these mechanisms could have influenced the presentation of PE in our patient during the luteal phase when E and P levels are high. The OCP administered (Yasmin Diario® o Diane 35 Diario®) has a negative feed-back mechanism on hypophysis hormones lowering endogenous E’s levels throughout the menstrual cycle, including the pre-ovulation peak. The subcutaneous implant of etonogestrel (Implanon®, Organon Española S.A. Laboratories, Madrid, Spain) has a slow and sustained release of a P derivate which inhibits endogenous progesterone levels throughout the menstrual cycle, including the luteal phase.

Based on a careful study of this patient’s clinical history we speculate that both the year-long effectiveness of the aerosolized ampicillin treatment and the 7-month effectiveness of the treatment with the first OCP (Yasmin Diario®) may be explained by the patient’s young age. The first two years after the onset of menarche are characterized by anovulatoy cycles which cause irregular E and P patterns during the menstrual cycle. After these two years, the patient probably started experiencing regular ovulatory cycles which would have normalized her E and P levels across the normal menstrual cycle. The OCP prescribed (Yasmin Diario® and Diane 35 Diario®) only contain E, thus, they could not be effective in this particular case because our patient had the PE always during the luteal phase, rather than the preovulatory, or immediately before the menstrual phase of the cycle when the E are high. We think the effectiveness of the implant, inserted around two years and a half after menarche, was secondary to the reduction of E and P levels caused by the administration of exogenous P throughout the entire menstrual cycle, including the luteal phase, while withholding administration of exogenous E.

The efficacy of ovulatory inhibition with this type of implant varies from woman to woman across time, diminishing by 10% to up to 50% between year 2 and 5 after insertion. In our case, a substantial loss of efficacy could explain the patient’s new acute PE episode after the first two years post-insertion and the disappearance of clinical symptoms again after the insertion of a new implant.

The genotype of this patient (R334W, class IV mutation, and Q890X, class I mutation) is uncommon and, in this case, associated with a severe expression of the disease with pancreatic sufficiency, numerous episodes of acute pancreatitis, and severe pulmonary disease with frequent PE. Sutton et al. [4] analyzed the United States CF Foundation Patient Registry for a retrospective study of LF decline and rate of PE changes in CF before and after puberty*.* They concluded that whereas males had lower rate of PE than females, there was no association between annual frequency of PE and race, pancreatic insufficiency, or genotype. Further research is necessary to shed light on the relationship between CF genotype and the effect of female hormones on morbidity/mortality in women.

The effect of female hormones on women with CF is a complex issue given that women have different PE recurrence patterns according to the different phases of the menstrual cycle. Thus, studying the history of PE pattern is essential in order to prescribe one contraceptive method over another (with exogenous E, P, or a combination of both) according to the expression of clinical symptoms of each patient.

## Conclusion

Pulmonary epithelium is a target for female hormones, including E and P. Their effects on lung function, inflammation, and immune responses may impair MCC and are likely to partially explain the increase in PE frequency observed in women with CF. The case presented here underlines the likely contribution of female hormones to the sex differential in morbidity/mortality in patients with CF. This case supports hormonal manipulation as a potential highly effective therapy for women with CF.

## Consent

Written informed consent was obtained from the patient for publication of this Case report and any accompanying images. A copy of the written consent is available for review by the Editor-in-Chief of this journal.

## Abbreviations

CF: Cystic fibrosis
LF: Lung function
PE: Pulmonary exacerbations
E: Estrogens
*P. aeruginosa*: *Pseudomonas aeruginosa*
ASL: Airway surface liquid
CFTR: Cystic fibrosis transmembrane conductance regulator
P: Progesterone
OCP: Oral contraceptive pill
AP: Acute pancreatitis
*S. aureus*: *Staphylococcus aureus*
FEV_1_: Forced expired volume in 1 second
FVC: Forced vital capacity.
